# Factors Linked to Female Genital Mutilation Practice Among Women Living In Alungu Village of Mandera County, Kenya

**DOI:** 10.24248/eahrj.v7i1.716

**Published:** 2023-07-12

**Authors:** Mohammed Mohammud Sheikh, Joyce Jebet Cheptum, Irene Gacheri Mageto

**Affiliations:** a Mandera County Hospital, Mandera, Kenya; b National Defence University – Kenya, Nairobi, Kenya; c Department of Nursing Sciences, University of Nairobi, Nairobi Kenya

## Abstract

**Background:**

Female Genital Mutilation/Cutting (FGM/C) is a harmful traditional practice with severe health complications, deeply rooted in many sub-Saharan African countries. In Kenya, the prevalence of FGM/C is 15% in women aged between 15 and 49 years. The Kenyan Somalis practice FGM/C with a prevalence above 90%. FGM/C practice continues to persist in Alungu village, Mandera County in the North Eastern of Kenya despite efforts by anti-FGM programs. However, the underlying factors behind FGM practice in the area have not been explored. Objective: To assess factors contributing to female genital mutilation practice among women living in Alungu village of Mandera County, Kenya.

**Methods and materials:**

This study utilised a descriptive cross sectional design. The study population was women of reproductive age (from 18 to 49 years) who resided in Alungu village in Mandera County, Kenya. A study sample of 98 women was selected using simple random sampling technique. Data was collected using a researcher-administered questionnaire and analysed using the Statistical Package for Social Science (SPSS).

**Results:**

Most of the respondents were aged 35 – 44 (45.8%), married (100%), had no formal education (74.7%) and had no formal employment (89.2%). All participants agreed that traditional beliefs, customs and rite of passage to womanhood contributed to FGM, 90.4% of the participants acknowledged that FGM is a symbol of ethnic identity and inclusivity. Factors affecting prevention of and response to FGM were low involvement of women in anti-FGM programs (91.6%); support for FGM by local leaders and elders (100%); failure by authorities to take action against those perpetuating FGM (100%); indifference to FGM practice continuation among local religious and political leaders (96.4%) and poor enforcement of existing laws against FGM (100%).

**Conclusion:**

A wide range of socio-cultural factors did contribute to FGM practice among women living in Alungu village, Mandera County.

## BACKGROUND

Female genital mutilation (FGM) also known as female genital cutting (FGC) or female circumcision (FC) refers to the partial or full removal of external female genitalia or other intentional injury to the female genitals for non-medical reasons.^1^ The World Health Organization (WHO) estimates that more than 200 million women and girls alive today around the world have undergone female genital mutilation and/or cutting. It is further estimated that, globally, more than three million girls are at risk of undergoing FGM every year.^1^ The practice is mainly concentrated in the Western, Eastern, and North-Eastern regions of Africa, in some countries in the Middle East and Asia, as well as among migrants from these areas.^1^ FGM is highly prevalent in Africa with data from WHO and UNICEF indicating that over 90% of the 30 FGM high prevalence countries were in the African region. FGM prevalence rate in Africa range from 35 to 40 percent is way above the global prevalence rate of below 10%. Worse still, in some African communities, the prevalence of FGM was as high as over 90% denoting a concern that required urgent.^1,2^

Data from censuses, household and hospital records indicate that FGM constitutes a massive global health challenge particularly in light of the high burden of FGM in Africa, the Middle East and Asia where the practice is concentrated.^1^ In Africa, more than 33 percent of girls undergo FGM yearly in majority of African countries. The practice of FGM is strongly bound by cultural norms and traditions, such as the rite of passage into womanhood.^2,3^ The removal of parts of female genitalia is an ancient tradition in many parts of Africa and its practice world over has persisted despite existing political and legal penalties.^4^

FGM practice has been linked to adverse physical and psychological consequences like post-traumatic stress disorder and affective disorders.^5^ The physical consequences can be short term for example bleeding, pain, acute urine retention or long term for example keloids, epidermal cysts and chronic urinary tract infections.^6^ Women who have undergone FGM have experienced obstetric complications for example obstructed labour, extensive perineal tears and operative births.^7,8^ FGM also leads to sexual complications.^9–11^

In sub-Saharan Africa (SSA), majority of the countries still practice FGM which is marked with rituals and celebrations and is regarded as being an integral part of girls' social development. In addition, FGM practice in SSA is deeply embedded largely on account of cultural and social norms of communities and there is immense social pressure on all young girls to conform to this practice.^2^ Studies done in SSA indicate that girls who fail to undergo FGM are likely to be punished or penalized while others are isolated, stigmatized and despised by not being married by men from their communities. This forces them to leave their communities or they are forced by their parents to undergo the practice.^12^

Ignorance of sexual and reproductive health, particularly inadequate knowledge on the negative health impacts of the practice greatly contributes to the persistent practice of FGM among different communities in SSA, the East Africa region included. Studies conducted in Uganda, Tanzania and Kenya showed that remote communities still believed that FGM promoted health and personal hygiene. In addition, the smooth surface was said to be pleasing for both the man and the woman.^4,13,14^ Among factors cited to contribute to this harmful practice in the East African region include its social acceptance, low level of awareness about its harmful impact on the health of women and girls, cultural beliefs and traditions, ignorance on women's reproductive health issues among local communities and beliefs that FGM leads to increased pleasure for male partners, enhanced fertility and improved marriage prospects.^4,15,16^

The prevalence of FGM in Kenya among women of reproductive age is at 15 percent with the highest prevalence in the northern part of Kenya at 43 percent among girls aged 10 to 14 years.^17,18^ FGM rates in Kenya vary considerably by location from rates of less than 20% in Central, Coast, Nairobi and Western regions to rates of 20% to 60% in Rift Valley, Nyanza and Eastern regions to as high as over 95% in North Eastern. More than 80 per cent of the FGM are performed by traditional excisors.^18^ Despite the general decline of female genital mutilation, the prevalence of FGM remains high in Kenya's North Eastern communities such as the Somali at 94% and Samburu at 86%. This has been attributed to low levels of knowledge on the adverse effects and impact of FGM on women and girls.^2^ Despite laws in place prohibiting FGM in Kenya, evidence from Alungu village in Mandera County showed that the community still practiced FGM among girls aged 7 to 15 years.^19^ The evidence indicated that one in every three girls from the village had either undergone the cut or was waiting to undergo the cut. In light of this serious situation, the current study sought to explore the factors linked to female genital mutilation practice among women living in Alungu village of Mandera County, Kenya. Elimination of FGM incidences is an important strategy towards the achievement of Sustainable Development Goals (SDGs) on health.

The fight against FGM also contributes to international community's efforts towards achieving gender equality and empowering all women and girls by eliminating all harmful practices such as child marriages, early or forced marriages and FGM.

## METHODOLOGY

### Study design

The study utilised a community based cross-sectional descriptive research design which employed quantitative approach through the use of interviewer administered questionnaire.

### Study Site

The study was carried out in Alungu village in Mandera County in the North Eastern Part of Kenya. Mandera County borders Ethiopia to the North, Somalia to the East and Wajir County to the South and South West and is characterized by low lying rocky hills located on the plains that rise gradually from 400 meters above sea level. The rest of topography is low lying, characterized by dense vegetation with thorny shrubs of savannah type found along foots of isolated hills. The major economic activity in Mandera County is pastoral with farming activities being undertaken along Daua River. The County has a population of 1,025,756 people according to the 2009 census (KNBS, 2016). Alungu village lies within Lafey Sub-County of Mandera County, Kenya.

### Study population

The study targeted women of reproductive aged between 18 and 49 years who resided in Alungu village in Mandera County and consented to the study.

### Sample size and sampling procedure

The study sample size was calculated using Yamane's formula.

n=N/1+N(e)^2^

150/1+150(0.05)^2^

150/1+150(0.0025)

150/1+0.375

150/1.375

n = 109

Where;

N is the estimated population size =150 (Number of women of reproductive age in Alungu village).

E is the margin error which is 0.05

Hence, the study sample size comprised of 109 women who live in Alungu village, Mandera County.

To obtain the study sample, simple random sampling technique was employed, where a random number generator was used to generate 109 numbers. This ensured that all the participants had an equal chance of participating in the study. This sampling was done during meeting breaks and the interviews were carried out after the meeting. The list of women was made and each woman was allocated a number. Using the random number generator, a respondent who had the number was picked.

### Data Collection Procedure

Researcher approached the women residing in Alungu village, individually, during their weekly women group meetings that occurred at Mama Yarey's Meeting Room within the village to request for their participation in the study. The researcher targeted the women during their meetings' break intervals. The brief encounters did not last for more than 5 minutes. During these brief encounters, the researcher highlighted important points about the study; emphasized the study's selection criteria and disclosed that he and the lady chaperone were available at the local health facility's Counselling Room for further details about the study. The inclusion criteria was women of reproductive age who had resided in the study site for not less than one year and those who consented to the study. Women who met the inclusion criteria met the principal researcher and the lady Chaperone in the specified area at their convenience for in-depth information and procedure of participation.

### Data Quality Assurance

A pre-test was carried out to ensure the validity and reliability of the research tool. The data collection exercise entailed the researcher asking the respondents the questions as contained in the research tool and noting down their responses. Ethics were observed throughout the process of research to avoid introduction of biases. Once the study participants responded to the questionnaires, the researcher scrutinized them for completeness before receiving them. Data analysis process was conducted systematically to avoid any alterations.

### Data Analysis, Presentation and Storage

The filled-in questionnaires were then stored safely under lock and key in readiness for data entry and analysis. The data collection exercise took three weeks.

The quantitative data generated from the closed ended questions were analysed through descriptive statistics using the Statistical Package for Social Science (SPSS, version 24) and presented through percentages and frequencies. The study results were presented in tables, graphs and charts, as appropriate.

### Ethical Approval

Ethical approval was sought from the Kenyatta National Hospital - University of Nairobi Ethics Research Committee (ERC), ethical approval number UP618/07/2021 and permission was sought from relevant authorities at the Mandera County Offices and the local authority.

Given the cultural sensitivity of the study subject and the fact that it was culturally inappropriate for the principal researcher (being a man) to interview the respondents (who were women) by himself, the principal researcher utilized a lady chaperone during the data collection process. The lady chaperone was a reproductive health counsellor at the local health facility from where the data collection exercise was carried out.

## RESULTS

### Response Rate

From the interviews held, adequate responses from 83 respondents were obtained, translating into a response rate of 76.1%

### Respondents' Profile

From the findings, 45.8% (n=38) of the respondents were aged 35-44 years. Most (74.7%, n=62) of the respondents had no formal education and majority (89.2%, n=74) were unemployed. All the respondents were married, from Muslim religion and from Somali ethnicity. Most (81.9%, n = 68) of the respondents had 5 - 8 children. (Table 1).

**TABLE 1:** Demographic Characteristics of Respondents

Variable	Frequency (n)	Percentage (%)
Age group		
18 - 24	8	9.6
25 - 34	25	30.1
35 - 44	38	45.8
45 - 49	12	9.6
Education level		
No formal education	62	74.7
Primary	17	20.5
Secondary	4	4.8
Marital status		
Married	83	100
Occupation		
Formal employment	2	2.4
Self -Employed	7	8.4
Not employed	74	89.2
Number of children		
1 - 4	5	6.1
5 - 8	68	81.9
More than 8	10	12
Household monthly income		
< Kshs. 5,000	21	25.3
Kshs. 5,000 - Kshs. 20,000	57	68.7
Above Kshs 20,000	5	6.0
Religion		
Muslim	83	100
Ethnicity		
Somali	81	97.6
Others	2	2.4

Majority of the respondents (96.4%, n=80) had undergone female genital mutilation. The results also revealed that the respondents underwent the “cut” at ages between 7 and 15 years, and in 86.7% of respondents said that FGM was performed by traditional practitioners.

### Socio-cultural factors and FGM practice

All the respondents unanimously agreed that traditional beliefs and customs did influence the practice of FGM in their community. To them, FGM practice was a traditional cultural event held in high regard among community members. This implied that traditional beliefs and customs were a leading factor that contributed to the FGM practice in the respondents' community. All the respondents concurred that indeed there was immense social pressure to conform to traditional values that supported FGM as a social norm in their community. The social pressure took the form of social isolation or exclusion, discrimination and not being perceived as a “complete” woman for those adolescent girls and women who refused to undergo FGM. This denoted that social pressure to conform to traditional values that supported FGM as a social norm in the respondents' community played a significant role in the perpetuation of the FGM practice among women residents of Alungu village.

Most of the study participants strongly agreed with the views that traditional beliefs and customs were the main drivers of FGM in their community. FGM was performed because it was considered an important part of their culture as cited by 96.4% (n=80) of the respondents. All respondents reported that FGM was considered a rite of passage for girls in their community and marked transition to womanhood. Among the respondents, 86.7% (n=72) said that FGM was done for beauty, hygiene and cleanliness with uncircumcised girls being considered unclean and unfeminine. Majority of respondents (89.2% (n = 74)) believed that FGM was an integral part of a woman's social status in their community.

Further, most of the respondents (94%) strongly agreed with the views that girls who refused to undergo FGM were likely to be socially isolated, penalized and excluded from the society. Nearly all respondents (97.6%) said that FGM was linked with increased chastity and marriageability enabling women to avoid promiscuity before and during marriage. All respondents agreed that religious beliefs played an important role in perpetuation of FGM. Over 90% of respondents said that female circumcision create a sense of attachment and identity and that males' support for FGM played an important role in its perpetuation.

### Factors Affecting Prevention and Response to FGM

Respondents (91.6%: n=76) said that lack of or low involvement of women in anti-FGM programs compromised the fight against the practice. All respondents viewed that the overt or covert support for FGM by local leaders and elders, and failure by authorities to take action against perpetrators contribute to perpetuation of FGM in their community. Majority of the respondents (96.4%: n=80) said that the indifference to the continued practice of FGM among local religious and political leaders helped perpetuate the practice (Table 2).

**TABLE 2:** Factors Affecting FGM Response and Prevention

Statements on factors affecting FGM response and prevention	Disagree		Agree	
	Freq.	%	Freq.	%
Lack of or low involvement of women in anti-FGM programs compromises the fight against the practice	7	8.4	76	91.6
The overt or covert support for FGM by local leaders and elders contributes to perpetuation of FGM in our community	0	0.0	83	100.0
Failure by authorities to take action against those perpetuating the FGM practice compromises existing anti-FGM efforts	0	0.0	83	100.0
The indifference to the continued practice of FGM among local religious and political leaders helps perpetuate the practice	3	3.6	80	96.4
Lack of or low involvement of women in anti-FGM programs compromises the fight against the practice	7	8.4	76	91.6
The overt or covert support for FGM by local leaders and elders contributes to perpetuation of FGM in our community	0	0.0	83	100.0
Failure by authorities to take action against those perpetuating the FGM practice compromises existing anti-FGM efforts	0	0.0	83	100.0
The indifference to the continued practice of FGM among local religious and political leaders helps perpetuate the practice	3	3.6	80	96.4
Women's general lack of voice and autonomy in their own reproductive health matters in our community also fosters the FGM practice	5	6.0	78	94.0
Patriarchal authority structures that permeate our community are also to blame for the continued perpetuation of FGM	2	2.4	81	97.6
Poor enforcement of existing laws against FGM also contributes to its continued practice	0	0.0	83	100.0
Failure by local leaders to perceive FGM as a women's rights issues and not a cultural one helps perpetuate the practice	9	10.8	74	89.2
Lack of support for efforts to fight the FGM practice among community leaders significantly impedes the eradication of FGM	1	1.2	82	98.8

## DISCUSSION

The findings this study are in line with what have been reported by other studies which observed that FGM was largely practiced in communities for conformity to cultural and traditional beliefs, and norms including FGM being perceived as rite of passage for girls to womanhood and that it enhance chastity and purity making girls more marriageable.^20,16,21^ Similar to the findings of this study, Reig Alcaraz et al^22^ and Ogoe^4^ also noted that the need to conform to traditional cultural beliefs that supported FGM remained a leading reason for the continued practice of FGM in many settings across the globe. The findings of this study support the observations made by Moranga^15^ which attributed the persistence of FGM practice in one of Kenya's communities to FGM being used as a symbol of ethnic identity, a community mobilization tool and it being associated with feminine beauty and cleanliness. Several other studies found that traditions, cultural beliefs and social norms are the leading driving forces behind continued prevalence of FGM.^23–25^

### Factors Affecting FGM Response and Prevention

The results of this study are in agreement with those of Abebe et al^25^ and Gele et al^26^ who identified lack of local women's involvement in anti-FGC programs, lack of suitable legal penalties for those who practiced FGC exacerbated the problem and support of FGM by community elders and persons in positions of authority as significant factors behind FGM's continuance. Similarly, Mohamud et al^27^ and Andarge^28^ cited unsupportive legal framework, lack of action from administrative officers against persons perpetuating the FGM practice and indifference to the continued practice of FGM among local administrative, religious and political figures as major contributors behind the perpetuation of the FGM practice. Studies by Channel et al., Muteshi and Omolase et al^12,29,30^ also attributed the continued practice of FGM in many jurisdictions to weak legal and administrative frameworks and reluctance of local authorities to cramp the practice.

## CONCLUSION

A wide range of socio-cultural factors contributed to FGM practice among women living in Alungu village, Mandera County including FGM being part of their traditional cultural beliefs, customs and social norms, a rite of passage to womanhood associated with chastity, purity and cleanliness among women, and FGM being a symbol of ethnic identity and inclusivity and women's lack of voice in their own reproductive health matters as dictated by culture.

Low involvement of women in anti-FGM programs, failure by authorities to take action against those perpetuating FGM, poor enforcement of existing laws against FGM, and presence of patriarchal authority structures pose a barrier to FGM response and prevention efforts.

## RECOMMENDATIONS

The households and community should have concerted efforts, at household and community levels by all parties concerned, to address socio-cultural barriers to eradication of FGM practice. The County government should increase efforts to secure full enforcement of existing laws against FGM practice and all parties aiding the perpetuation of the practice should be brought to book in line with the law of the land. Through local organization. It is paramount that anti-FGM initiatives pay due consideration to targeted communities' religious and cultural beliefs and seek to find an amicable way of addressing the FGM problem while respecting the concerned people's religious and cultural orientations.

Future studies are recommended on male partners within the Community to be interviewed about their views regarding FGM practice. In addition, elder Women at Menopausal age to also be interviewed. A qualitative study could be done to get a deeper understanding of why the practice is still going on despite its negative effects.

### Limitation

The study gathered data from a single village in Mandera County, Kenya. Thus, the findings may not be generalized to all other regions in the country. In addition, the small sample size may not be representative to allow for generalization of findings.

## References

[1] WHO. Female genital mutilation. Key Facts. WHO webpage.

[2] Equality AG. A Decade of Action to Achieve The UNICEF Approach to the Elimination of Female Genital Mutilation.

[3] UN. International Day of Zero Tolerance for Female Genital Mutilation. Tiedote. Published online 2016.

[4] Ogoe SE. Why does female genital mutilation persist? Examining the failed criminalization strategies in Africa and Canada. J Chem Inf Model. 2015;53(9).

[5] Mulongo P, Hollins Martin C, McAndrew S. The psychological impact of Female Genital Mutilation/Cutting (FGM/C) on girls/women's mental health: a narrative literature review. J Reprod Infant Psychol. Published online 2014. doi:10.1080/02646838.2014.949641

[6] Reisel D, Creighton SM. Long term health consequences of Female Genital Mutilation (FGM). Maturitas. Published online 2015. doi:10.1016/j.maturitas.2014.10.009 25466303

[7] Muchene KW, Mageto IG, Cheptum JJ. Knowledge and Attitude on Obstetric Effects of Female Genital Mutilation among Maasai Women in Maternity Ward at Loitokitok Sub-County Hospital, Kenya. Obstet Gynecol Int. Published online 2018. doi:10.1155/2018/8418234 PMC6093056 30154858

[8] Berg RC, Underland V. The obstetric consequences of female genital mutilation/cutting: a systematic review and meta-analysis. Obstet Gynecol Int. Published online 2013. doi:10.1155/2013/496564 PMC3710629 23878544

[9] Rouzi AA, Berg RC, Sahly N, Alkafy S, Alzaban F, Abduljabbar H. Effects of female genital mutilation/cutting on the sexual function of Sudanese women: a cross-sectional study. Am J Obstet Gynecol. Published online 2017. doi:10.1016/j.ajog.2017.02.044 28267442

[10] Esho T, Kimani S, Nyamongo I, et al. The “heat” goes away: Sexual disorders of married women with female genital mutilation/cutting in Kenya. Reprod Health. 2017;14(1). doi:10.1186/s12978-017-0433-z PMC5712182 29197397

[11] Biglu MH, Farnam A, Abotalebi P, Biglu S, Ghavami M. Effect of female genital mutilation/cutting on sexual functions. Sex Reprod Healthc. Published online 2016. doi:10.1016/j.srhc.2016.07.002 27938869

[12] Channel M, Nancy S, Kanayo O. A review of the underlying factors influencing female genital mutilation in Africa. Stud Ethno-Medicine. 2016;10(3). doi:10.1080/09735070.2016.11905505

[13] Ahinkorah BO, Hagan JE, Ameyaw EK, et al. Socioeconomic and demographic determinants of female genital mutilation in sub-Saharan Africa: analysis of data from demographic and health surveys. Reprod Health. 2020;17(1). doi:10.1186/s12978-020-01015-5 PMC7584098 33092624

[14] Kimani S, Kabiru CW, Muteshi J, Guyo J. Exploring barriers to seeking health care among Kenyan Somali women with female genital mutilation: A qualitative study. BMC Int Health Hum Rights. 2020;20(1). doi:10.1186/s12914-020-0222-6 PMC6986153 31992317

[15] Everline MB, of Nairobi U. Factors Influencing the Practice of Female Genital Mutilation in Kenya: a Case Study of Gachuba Division, Nyamira County By Everline Bosibori Moranga a Research Project Submitted in Partial Fulfillment of the Requirement for the Award of Masters of Arts D. 2014;(May).

[16] Chidera E. What Factors Influence the Persistence of Female Genital Mutilation in Nigeria? A Systematic Review. J Trop Dis. 2018;06(01). doi:10.4172/2473-3350.1000256

[17] KNBS, ICF. Kenya Demographic and Health Survey 2022. Key Indicators Report. Kenya Natl Bur Stat Minist Heal. 2023;5(1):1–105. https://dhsprogram.com/pubs/pdf/PR143/PR143.pdf

[18] KDHS. 2014 DEMOGRAPHIC AND HEALTH SURVEY. Kenya Natl Bur Stat. Published online 2014.

[19] Ibrahim Mohamed H. Factors Promoting the Practice of Female Genital Mutilation among Women Seeking Reproductive, Maternal, Neonatal and Child Health Services at El-Wak Sub-County Hospital in Mandera County. SSRN Electron J. Published online 2021. doi:10.2139/ssrn.3835774

[20] Andro A, Lesclingand M. Female Genital Mutilation. Overview and Current Knowledge. Population (Paris). 2016;71(2).

[21] Kimani S, Kabiru CW, Muteshi J, Guyo J. Female genital mutilation/cutting: Emerging factors sustaining medicalization related changes in selected Kenyan communities. PLoS One. 2020;15(3). doi:10.1371/journal.pone.0228410 PMC7051066 32119680

[22] Reig Alcaraz M, Siles González J, Solano Ruiz C. Attitudes towards female genital mutilation: An integrative review. Int Nurs Rev. 2014;61(1). doi:10.1111/inr.12070 24237176

[23] Arora KS, Jacobs AJ. Female genital alteration: A compromise solution. J Med Ethics. 2016;42(3). doi:10.1136/medethics-2014-102375 26902479

[24] Berg RC, Denison E, Fretheim A. Factors Promoting and Hindering the Practice of Female Genital Mutilation/Cutting (FGM/C).; 2010. 29320107

[25] Abebe S, Dessalegn M, Hailu Y, Makonnen M. Prevalence and barriers to ending female genital cutting: The case of afar and amhara regions of Ethiopia. Int J Environ Res Public Health. 2020;17(21). doi:10.3390/ijerph17217960 PMC7663154 33138238

[26] Gele AA, Bø BP, Sundby J. Attitudes toward Female Circumcision among Men and Women in Two Districts in Somalia: Is It Time to Rethink Our Eradication Strategy in Somalia? Obstet Gynecol Int. 2013;2013. doi:10.1155/2013/312734 PMC3654358 23710186

[27] Mohamud M, Kaba M, Tamire M. Assessment of barriers of behavioral change to stop FGM practice among women of Kebri Beyah District, Somali Regional State, Eastern Ethiopia. Glob J Med Res K Interdiscip. 2016;16(6).

[28] Andarge MY. The Difficulties of Ending Female Genital Mutilation (FGM): Case of Afar Pastoralist Communities in Ethiopia. Int Inst Soc Stud. Published online 2014.

[29] Muteshi J. Root causes and persistent challenges in accelerating the abandonment of FGM/C. Evid to End FGM/C. 2016;(71st General Assembly).

[30] Omolase CO, Akinsanya OO, Faturoti SO, Omotayo RS, Omolase BO. Attitudes towards female genital cutting among pregnant women in Owo, Nigeria. South African Fam Pract. 2012;54(4). doi:10.1080/20786204.2012.10874250

